# Long-term disease-free survival in advanced melanomas treated with nitrosoureas: mechanisms and new perspectives

**DOI:** 10.1186/1471-2407-5-147

**Published:** 2005-11-15

**Authors:** Xavier Durando, Emilie Thivat, Michel D'Incan, Anne Sinsard, Jean-Claude Madelmont, Philippe Chollet

**Affiliations:** 1 Medical Oncology Unit, Centre Jean Perrin, Clermont-Ferrand, France; 2 Dermatology Department, Hôtel-Dieu, Clermont-Ferrand, France; 3 INSERM U484, Clermont-Ferrand, France

## Abstract

**Background:**

Median survival of metastatic malignant melanoma is 6.0 to 7.5 months, with a 5-year survival of ~6.0%. Although long-term complete remissions are rare, few reports describe cases after chemotherapy. Fifty-three patients with metastatic melanoma were treated with Cystemustine, a chloroethyl nitrosourea (CENU) (60 or 90 mg/m^2^).

**Case presentation:**

We describe 5 cases, presenting with complete response with long-term disease-free survival of long-term remission of 14, 12, 9, 7 and 6 years after Cystemustine therapy alone.

**Conclusion:**

Long-term survival has already been described in literature, but in all cases they have been obtained after chemotherapy associated with or followed by surgery. But despite these noteworthy and encouraging but also rare results, it appears essential to increase cystemustine efficiency.

## Background

Melanoma has become an important public health issue because of its rising incidence in the Caucasian population. Even though, most cases are cured by surgery alone in the early stages of the disease, advanced melanoma has a poor prognosis. Therapeutic strategies of metastatic malignant melanoma are based on multiple treatment including chemotherapy (dacarbazine, platinum analogs, chloronitrosoureas (CENU), vinca alkaloïds or taxanes) and immunotherapy (interferon α, interleukine 2), both as single agents or in association.

Median survival of metastatic malignant melanoma is 6.0 to 7.5 months, with a 5-year survival of approximately 6% [1]. Although long-term complete remissions are rare, some authors have reported cases after chemotherapy treatment [2-6].

Cystemustine {*N'*-(2-chloroethyl)-*N*- [2-(methyl sulphonyl)ethyl]-*N'*-nitrosourea} is a CENU derived from 2-chloro-ethylnitroso-carbamoylcysteamine (CCNC); it is synthesised in INSERM U484, Clermont-Ferrand [7]. This new compound has demonstrated an equivalent and often better chemotherapeutic index for solid tumours than other chloroethylating agents currently in use [8].

We describe 5 cases treated with Cystemustine (60 or 90 mg/m^2^) for metastatic melanoma showing complete responses with long-term survival (survival time 24 months or longer).

## Case Presentation

A series of 53 patients has been treated with Cystemustine administered as a 15 min iv infusion every 2 weeks. 30 patients were included in a phase II national and multicentric study, whereas other 23 patients received a Cystemustine compassionate treatment.

In the phase II study, patients provided written informed consent after they were informed about the objectives of the study. The protocol designs and relative modifications were fully approved by the ethic committee. The patients received Cystemustine 60 or 90 mg/m^2^. The median number of Cystemustine cycles was 3 (range, 2 to 7 cycles). 28 out of 30 patients were assessed for disease response (2 patients were included without measurable disease according to World Health Organisation (WHO) criteria. The overall response rate was 17.9%, with 3 complete responses and 2 partial responses. Two patients showing complete response had been treated with Cystemustine 90 mg/m^2^, the third patient received Cystemustine 60 mg/m^2^.

Patients given "compassionate treatment" received 60 mg/m^2 ^of Cystemustine. The median number of Cystemustine cycles was 3 (range, 1 to 27 cycles). Among these 23 patients, 15 were assessed for disease response (i.e. patients with measurable disease according to WHO criteria). Four complete and two partial responses were observed. All patients were treated with Cystemustine 60 mg/m^2^.

Among these 53 patients, 5 complete responses with long-term survival occurred. In 4 cases, complete responses were obtained after Cystemustine treatment alone, whereas, for the fifth patient, one of the metastatic lesions was excised at the early beginning of the treatment. The characteristics of these 5 patients are summarised in the "patient characteristics" table 1.

**Table 1:** Patient characteristics.

**Patient**	**1**	**2**	**3**	**4**	**5**
**Patient age/disease discovery (year)**	55	33	63	44	43
**Primary disease date**	April 1981	August 1985	January 1984	January 1997	August 1993
**Primary disease location**	Right ankle	Left calf	Left ankle	Left arm	Left thorax
**Clarck index**	III	V	IV	III	III
**First recurrence date**	March 1988	June 1986	April 1987	January 1997	March 1998
**Previous treatment for advanced ** **disease (before cystemustine)**	None	Dacarbazine Vindesine (6 cycles) Dacarbazine Vinblastine (6 cycles) Dacarbazine Interferon α 2a (6 cycles)	Interferon α 2a Radiotherapy	None	Dacarbazine (8 cycles)
**Treatment start date**	March 1988	May 1990	August 1990	February 1997	Novembre 1998
**Custemustine dose (mg/m^2^)**	90	60	90	90	60
**Metastatic sites**	Popliteal mass Lymph node	Lung Bone	Lymph node	Lymph node Sub-cutaneous	Lung
**Treatment concomitant to Cystemustine**	Surgery (March 1988)	None	None	None	None
**Recurrence of the disease**	None	None	None	None	None
**Date of death**	November 2000	Alive	March 2000	Alive	Alive
**Disease free survival (years)**	12	14	10	7	6

**Patient 1 **was a 55 year woman presented with a level III Clark right ankle malignant melanoma excised on April 1981. In 1988, she displayed an inoperable popliteal mass associated with a large right inguinal adenopathy. Between March and August 1988, she received 13 cycles of Cystemustine 90 mg/m^2^. CR was documented by clinical and echographic exams in August 1988. The main toxicities of treatment were nausea, vomiting and thrombopenia. These last ones have led to dose reduction and treatment delay. This patient died of stroke on November 2000 without any recurrence of the disease.

**Patient 2 **was a 33 year woman presented with a level IV Clark left calf malignant melanoma excised on August 1985. Between 1986 and 1990, several relapse of melanoma were diagnosed. The patient has been successively treated with vindesine/dacarbazine, vindesine/dacarbazine, and dacarbazine/interferon α. Hormonotherapy with tamoxifene was then introduced with close clinical follow-up. In 1990, she presented with right tibiae metastasis and two pulmonary lesions. Treatment with Cystemustine 60 mg/m^2 ^was introduced for 18 cycles. After 6 cycles, complete regression of pulmonary lesions was observed, but bone lesions were always present in spite of a significant improvement. Tumour assessment after 17 cycles revealed CR to treatment. The main toxicity was haematological, with neutropenia and thrombopenia, that necessitated course delay and dose reduction.

**Patient 3 **was a 63 year woman presenting with Clark level IV malignant melanoma on left ankle, excised in January 1984. Three years later, tumour recurrence was diagnosed on the left calf. An adjuvant treatment with interferon was introduced (but interrupted after the first infusion because of an allergic reaction), followed by 3 × 18 Gy cycles of radiotherapy in June 1987. In August 1988, a node was detected at the lower part of the left popliteal pit, and was excised in December 1988. In September 1990, supra-clavicular and lombo-aortic nodes were detected. The patient received 11 cycles of Cystemustine 90 mg/m^2^, and a CR was obtained after 8 cycles. Haematological toxicities occurred with severe thrombopenia, leading to course delays and platelet transfusion after the 8^th ^infusion. Except for the haematological toxicity, treatment was well tolerated. However, a systematic exploration of respiratory function in January 2000 showed a severe hypoxia and confirmed the diminution of carbon monoxide capacity found 5 years earlier.

In July 1998, myelodisplasic syndrome was diagnosed. She died in March 2000 during severe aplasia, nevertheless her meanoma was still in complete remission for 9 years and a month.

**Patient 4 **was a 44 year male presenting Clark level III malignant melanoma located on left arm. Tumour excision was performed on January 1997 and completed with a node dissection. One node of 11 removed was metastatic. Imaging assessment found also a superficial 2 cm left axillary node as well as 2 – 3 infracentimetric metastatic subcutaneous nodes in the chest wall. The patient received 6 cycles of Cystemustine 90 mg/m^2^. Target size decreased after 2 cycles, and the patient was CR at the end of treatment. No relevant toxicity was reported, except an episode of febrile aplasia after the 6^th ^cycle leading to red blood cells and platelets transfusion.

**Patient 5 **was a 43 year male presenting with a Clark level III cutaneous thoracic melanoma lesion excised on August 1993. Surgery was completed by a wide local excision margin of 3 cm of the scar. Disease assessment imaging revealed no metastasis. The patient was then followed every 3 months.

Several pulmonary node metastasis were found in March 1998. The patient received 8 cycles of dacarbazine, and because of new disease progression, he received six cycles of Cystemustine 60 mg/m^2 ^every 2 weeks. The patient was considered in complete remission after 2 cycles of Cystemustine. No significant toxicity was reported during the treatment.

Among these five patients, three remain in long-term remission for 14, 7 and 6 years after Cystemustine monotherapy. Of the other 2 patients, one (patient 1) died from a stroke after a 12 years survival without disease recurrence. The second (patient 3) presented with a myelodysplastic syndrome treated with cytarabine. This patient died in March 2000 from severe aplasia, and at this time her melanoma was still in complete remission after 9 years.

## Conclusion

Although some reports had already described long-term survival in patients with metastatic melanoma treated with chemotherapy, most of these results were observed for patients treated with chemotherapy in association, or with chemotherapy alone followed by surgery. In a phase III study, 580 patients were treated with dacarbazine (Deticene^®^) alone or in association with carmustine, lomustine, vincristine or an hydroxyurea. Hill *et al*. [2] reported 8 patients, with complete response who survived 6 years after the treatment. Ahmann *et al*. [9] gave results for 15 clinical studies. Among the 503 patients, 6 presented with complete response and long term survival of seven years or longer. Four patients have been treated with a semustine based regimen, one with a combination of vinblastine, bleomycin and cisplatin, and the last one with dianthydrogalacticol.

Petit *et al*. [5] described 5 out of 160 patients who went into long-term remission 7 years after fotemustine chemotherapy followed by surgery. In Samuel *et al*. [6], 40 patients with symptomatic metastatic melanoma were treated with procarbazine, vincristine and lomustine (POC). Among them, two remained in complete remission at 6 and 6.5 years. Berd *et al*. [3] treated 147 patients with a combination of carmustine, dacarbazine and cisplatin. Among the 17 complete response, 7 patients presented with long-term survival (19 to 82 months).

Coates *et al*. also described the case of 8 patients who remain in long-term remission 4 to 15 years after chemotherapy for visceral metastatic melanoma among some 1100 patients with visceral melanoma who have received chemotherapy, almost always with single agent dacarbazine or a nitrosourea treatments. For these patients, the mechanism remains uncertain, but the author suggested that chemotherapeutic agents can cause mutations that allow expression of antigenicity in tumour cells [4].

Despite these noteworthy and encouraging but also rare results, it appears essential to increase cystemustine efficiency. The main antitumor target of CENU is DNA with generation of *O*^6^-chloroethylguanine and cross-linking with cDNA strands [10]. The MGMT (O6-methylguanine-DNA methyltransferase) protein removes O6-alkylguanine by accepting the alkyl group on the cystein residue of its active site. MGMT level varies considerably in normal and tumor cells, and cells that exhibit a low MGMT level are more sensitive to CENU [11]. Methionine restriction is known to inhibit growth of human and animal tumor (*in vitro*, and *in vivo*) [12]. It decreases MGMT mRNA and activity in tumoral cells [13]. Furthermore, the potentiating effect of methionine depletion on Cystemustine treatment has been shown in B16 melanoma-bearing mice [14]. On the basis of these previous experimental results, we have initiated a phase-I clinical trial of dietary methionine restriction, in association with Cystemustine treatment for adults with recurrent metastatic melanomas.

In conclusion, we described case of five out 53 patients with metastatic melanoma, treated with Cystemustine, 60 or 90 mg/m^2^, and presented with complete response and long-term disease-free survival. Despite these encouraging results, it appears crucial to increase cystemustine efficiency.

## Competing interests

The author(s) declare that they have no competing interests.

## Author's contributions

XD was involved in patient treatment and participated in writing the manuscript.

ET was responsible for data management, and participated in writing the manuscript.

MD'I, AS and PC were involved in patient treatment and participated in drafting the manuscript.

JCM has provided Cystemustine and participated in drafting the manuscript.

All authors read and approved the final manuscript.

## Pre-publication history

The pre-publication history for this paper can be accessed here:


